# High-Performance Coaxial Counter-Rotating Triboelectric Nanogenerator with Lift–Drag Hybrid Blades for Wind Energy Harvesting

**DOI:** 10.3390/nano14070598

**Published:** 2024-03-28

**Authors:** Fei Yan, Junhao Zhao, Fangming Li, Yiyao Chu, Hengxu Du, Minzheng Sun, Ziyue Xi, Taili Du, Minyi Xu

**Affiliations:** 1 Dalian Key Lab of Marine Micro/Nano Energy and Self-Powered System, Marine Engineering College, Dalian Maritime University, Dalian 116026, China; 2 Collaborative Innovation Research Institute of Autonomous Ship, Dalian Maritime University, Dalian 116026, China; 3 State Key Laboratory of Maritime Technology and Safety, Dalian 116026, China

**Keywords:** coaxial counter-rotating, distributed wireless sensor nodes, triboelectric nanogenerator, wind energy harvesting, Internet of Things

## Abstract

Wind energy holds potential for in-situ powering large-scale distributed wireless sensor nodes (WSNs) in the Internet of Things (IoT) era. To achieve high performance in wind energy harvesting, a coaxial counter-rotating triboelectric nanogenerator with lift–drag hybrid blades, termed CCR-TENG, has been proposed. The CCR-TENG, which can work in non-contact and soft-contact modes, realizes low-speed wind energy harvesting through a combination of counter-clockwise rotating lift-type blades and clockwise rotating drag-type blades. Non-contact CCR-TENG realizes low-speed wind energy harvesting at wind speeds as low as 1 m/s. The output of a CCR-TENG, working in soft-contact mode, achieves 41% promotion with a maximum short-circuit current of 0.11 mA and a peak surface power density of 6.2 W/m^2^ with two TENGs connected in parallel. Furthermore, the power density per unit of wind speed achieves 746 mW/m^3^·s/m. Consequently, two fluorescent lamps were successfully illuminated and six temperature sensors were continuously lit by the CCR-TENG. The reported CCR-TENG significantly improves low-speed environmental wind energy utilization and demonstrates broad application prospects for in-situ power supply of distributed wireless transmission devices and sensors in the era of the IoT.

## 1. Introduction

Based on a large amount of low-power WSNs and wireless transmission devices, the IoT makes everything interconnected. It increases humanity’s progress in digitization, intelligence, and informatization by leaps and bounds [1]. The burgeoning demand for distributed wireless transmission devices/sensors, statistically supported in the billions, mandates a parallel escalation in distributed energy supply [2,3]. The supply is projected to double by 2035 and increase to 1 GW per day by 2050 [4]. The challenge of energizing the extensive array of hundreds of millions of distributed WSNs is formidable. Their limited lifespan constrains reliance on traditional batteries for power supply, coupled with the challenges and high replacement costs [5,6]. Burning fossil fuels generates significant energy but results in severe environmental pollution. Additionally, long-distance power transmission incurs substantial energy losses and increased costs. Moreover, distributed sensing devices require a small-scale, continuous, and efficient energy supply, a demand that large thermal power systems find challenging to meet. In contrast, the local collection of environmental energy to supply these nodes represents a more effective and feasible energy provision method [7]. Wind energy, one of the most prevalent and accessible environmental energy sources due to its wide distribution and green and renewable advantages, is expected to achieve large-scale development and application [8,9,10,11].

Various wind energy harvesting devices using different methods have been investigated, including those based on the law of electromagnetic induction, piezoelectric effect, triboelectric effect, and electrostatic coupling effect. Each method has made some progress in the field of wind energy harvesting [12,13,14,15]. Among these methods, the triboelectric nanogenerator (TENG) is a promising approach for distributed energy harvesting due to high power generation, low-cost processing, wide availability and selection of materials, relatively simple device configurations and high directional adaptability [16,17,18]. Even more, the rotating TENG (R-TENG), compared to other types of TENG structures, offers higher efficiency, continuity, and high electrical output [19,20].

The performance of the R-TENG has been further improved through a series of scientific research studies targeting various aspects. The breeze-driven soft-contact mode TENG (BD-TENG) proposed by Li et al. exhibits excellent power generation performance. With a peak output of 330 V, 7 μA and 137 μC in full-contact mode, the BD-TENG can illuminate 300 series-connected red and blue LEDs at a startup wind speed of 3.3 m/s [21]. While the full-contact mode achieves higher power generation performance, it also reduces device durability and increases the startup speed of the device. To reduce the friction between dielectric materials, Yong et al. proposed a soft-contact concentric dual-shaft TENG (D-TENG), and by improving the device structure and contact mode, the startup wind speed of D-TENG is only 2.2 m/s [22]. Han et al. developed a polarity-controlled and soft-triggered (PS) TENG that alternately attaches dielectric materials to the copper film of the generating conductor using soft-contact friction, realizing a high durability of 372,000 rotations with unchanged output [23]. The friction between dielectric materials produces a short-circuit current, reduces the rotational speed of the device and hinders the enhancement of the R-TENG’s power output. To reduce the friction between dielectric materials and enhance the relative rotational speed of the R-TENG, Lin et al. reported a NC-Mode free-rotating disk triboelectric nanogenerator (FRD-TENG), which induces power generation through a “pre-charging” dielectric material, producing an open-circuit voltage of 220 V [24]. Ma et al. proposed a geared coaxial counter-rotating TENG (CCR-TENG), which generates more than 15 times more electron energy than conventional devices through efficient motion mode conversion [25]. Although the above work has improved durability, startup performance, rotational performance, and output performance, respectively, it is still challenging to balance, reducing friction and increasing the output of the R-TENG. It is challenging for the R-TENG to simultaneously have high durability, low-speed startup, high rotational performance, high durability and high output performance.

To efficiently achieve low-speed wind energy harvesting, high wind energy recovery efficiency, and high-power output, inspired by the contra-rotating propellers [26,27,28] and the combined structure of vertical-axis wind turbines [29,30], a coaxial counter-rotating triboelectric nanogenerator with lift–drag hybrid blades (CCR-TENG) has been proposed in this study. The CCR-TENG combines the counter-clockwise rotating lift-type external blades and the clockwise rotating drag-type internal blades, along with the R-TENGs at both ends. CCR-TENG can work in two modes: non-contact mode (NC-Mode) and soft-contact mode (SC-Mode). NC-Mode offers better startup and rotational performance but suffers from charge decay over time, while SC-Mode provides better stability despite higher rotational resistance. In NC-Mode, CCR-TENG can start at ultra-low wind speeds of 1 m/s. In SC-Mode, CCR-TENG can produce a maximum short-circuit current of 0.11 mA, with a peak surface power density of 6.2 W/m^2^ and a peak volumetric power density of 746 mW/m^3^·s/m. It is capable of powering two 14 W fluorescent lamps. When the wind speed stabilizes at 6 m/s, the SC-Mode CCR-TENG can keep the six temperature sensors functioning by charging a 330 µF capacitor. In summary, the reported CCR-TENG presents a feasible solution for in-situ powering for large-scale distributed wireless sensor nodes (WSNs) in the Internet of Things (IoT) era.

## 2. Materials and Methods

CCR-TENG’s materials and manufacturing methods: CCR-TENG consists of two interconnected counter-rotating dual-turbine blades and two rotating triboelectric nanogenerators. The dual-turbine blades include internal blades and external blades. The rotating triboelectric nanogenerator comprises a first turntable and a second turntable, with the first turntable connected to the external blades to form a containment space. It is fixed to the external blades and rotates with them, while the combined assembly of the second turntable and the internal blades is placed within the containment space and rotates with the internal blades. The internal surface of the top and bottom of the first turntable frictionally contacts the external surface of the top and bottom of the second turntable, respectively. The top and bottom surfaces of the first turntable are each equipped with six equally sized circular protrusions, onto which 100 µm thick copper film is attached. The top and bottom surfaces of the second turntable are uniformly divided into six equally sized sectors, onto which 100 µm wide nylon and 100 µm thick polytetrafluoroethylene (PTFE) films are alternately attached as the friction materials.

The external blades in the experimental device have a vertical axis lift-type S1046 airfoil with three blades, and the internal blades have a two-blade Savonius drag-type airfoil. The diameter of the first turntable is 235 mm, the height of the external blades is 220 mm, the diameter of the second turntable is 180 mm, and the height of the internal blades is 197 mm. The device is 3D printed from ePLA-LW lightweight foam to reduce drag and improve startup capability. The resulting device had a total weight of 350 g. By placing different numbers of polyester fur friction materials on the first turntable and alternating nylon and PTFE film friction materials on the second turntable, two turntables undergo periodic coaxial counter-rotating motion under external wind conditions, inducing a charge on the copper film on the first turntable. The induced charges on the first turntable can be extracted through external wires coupled with conductive fabric or electric brush structures, producing different outputs based on the wind conditions.

Electricity measurement: CCR-TENG experiments were conducted in a wind tunnel with dimensions of 1 m (length) × 0.4 m (width) × 0.4 m (height). The wind speed varied from 1 m/s to 9 m/s. The blower is installed on the right end of the wind tunnel, and the inverter is adjusted to make wind speed by adjusting the speed of the blower. A data monitoring, collection and preprocessing system was constructed using LabVIEW software and NI acquisition cards to realize real-time monitoring, data collection and rough processing of the experimental data. The rotational speed of the turntable is measured by a non-contact, high-precision opto-digital tachometer UTI-T372. The measurement method consists of sticking reflective stickers on the internal and external blades, respectively, and using the tachometer to directly measure the rotational speed of the internal and external blades to obtain the rotational speeds of each, which can be added together to obtain the relative rotational speed of the CCR-TENG. Meanwhile, to test the accuracy of this measurement method, the frequency of the output current waveform of the CCR-TENG is measured by an electrostatic voltmeter. The relative rotational speed of the CCR-TENG can be obtained after calculation. Compared with the results of the tachometer, the error is at most 20 rpm, and the accuracy of the tachometer-measured rotational speed is well established. The output data of voltage, short-circuit current, and transferred charge quantity is measured by an electrostatic voltmeter (Keithley6514, Keithley Instruments, Inc., Cleveland, OH, USA). The output voltage of CCR-TENG is far beyond the range of Keithley 6514. We estimate the output voltage of CCR-TENG by measuring the current passing through external resistors. The CCR-TENG is connected in series with two 500 MΩ high-voltage resistors, and the current is obtained. By Ohm’s law, the output voltage of the CCR-TENG is obtained by multiplying the current with the resistance.

## 3. Results and Discussion

### 3.1. Structural Design and Working Principle

**Figure 1:**
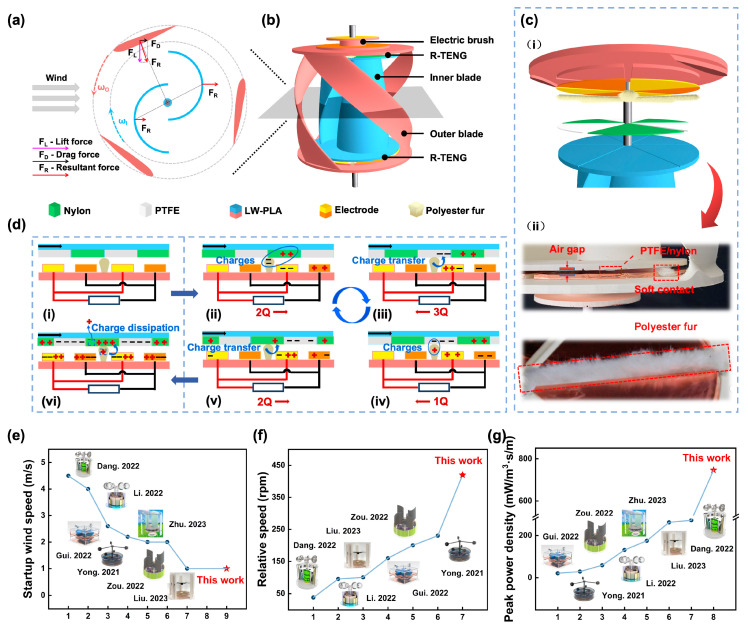
Structural design of the CCR-TENG. (**a**) Force analysis of a cross-section of the CCR-TENG; (**b**) Structural diagram of the CCR-TENG; (**c**) Schematic of SC-Mode of the CCR-TENG: (**i**) structural diagram, (**ii**) practical diagram; (**d**) Charge transfer diagram in SC-Mode of the CCR-TENG (The “+” and “−” in the picture indicate positive and negative charge); Comparison of (**e**) Startup wind speeds [22,33,34,35,36,37,38,39,40]; (**f**) Relative rotational speeds (at 5 m/s) [22,35,37,38,39,40]; and (**g**) Peak power density per unit wind speed between the CCR-TENG and other wind harvesting TENGs [22,35,36,37,38,39,40].

The CCR-TENG uses a structure in which the lift-type external blade and the drag-type internal blade are counter-rotating coaxially. Figure 1a shows the force analysis of a cross-section of CCR-TENG, in which the external blade wind wheel axis is located on the radial line of the blade circle, and the blade moves along the wing path to a position on the upwind side. Assuming that the wind enters from the left side, the pink vector $F_{L}$ represents the lift force acting on the blade, the black vector $F_{D}$ represents the drag force acting on the blade, and the red vector $F_{R}$ represents the resultant force of the lift force $F_{L}$ and the drag force $F_{D}$. The resultant force generates a torque on the wind turbine axis, which causes the external blade to rotate counterclockwise and the internal blade to rotate clockwise. According to the theoretical analysis, the forces on different cross sections of the internal and external blades are different, but always produce sufficient moment to realize coaxial counter-rotation. Video S1 shows CCR-TENG continuously powering two 14 W fluorescent lamps at a fixed wind speed of 4 m/s and records the coaxial counter-rotation effect of CCR-TENG at 0.5 times recording speed. The low relative rotational speed of CCR-TENG at this wind speed and the significant rotation of both the internal and external blades make it easy to observe the coaxial counter-rotating effect. The internal and external blades are designed as streamlined rotation types to prevent the blades from becoming stuck and unable to start at certain angles of attack in Figure 1b.

In this work, the coaxial counter-rotating structure is proposed to enhance the relative rotational speed of wind turbine blades during low-speed incoming wind. The sum of the rotational speeds of the internal and external blades determines the relative rotation speed of CCR-TENG. As shown in Equation (1), the resistance force $F_{D}$ experienced by the blade rotation exhibits an exponential relationship with the linear velocity v, indicating that the lower the rotational speed, the smaller the resistance force. The coaxial counter-rotating structure allows CCR-TENG to achieve a higher relative rotation speed while experiencing a significantly lower combined resistance force on the internal and external blades than the resistance force generated by a single blade rotating at the same relative speed. Thus, when facing low-speed wind, CCR-TENG encounters less resistance, freeing up more energy to increase the rotational speed of the wind turbine blades and resulting in a higher relative speed. Compared to a single-blade design, the coaxial counter-rotating structure reduces the overall resistance of the device during rotation under the same wind conditions, leading to a higher relative speed. The above are the key advantages of using the coaxial counter-rotating structure.

(1) $$F_{D}=\frac{1}{2}C_{D}A\rho v^{2}$$

where the drag force acting on the blades is represented by $F_{D}$, $C_{D}$ denotes the dimensionless drag coefficient, the contact area between the air and the blades is represented by A, the density of the fluid (air) is represented by ρ, and v represents the linear velocity of the blade tip.

In this design, the CCR-TENG can be classified as in non-contact mode (NC-Mode) and partially soft-contact mode (SC-Mode). The friction materials and the conductive copper film are initially uncharged in NC-Mode. Moreover, during the rotation of NC-TENG under wind conditions, there is no frictional charging between the friction materials. Before the experiment, the friction materials are “pre-charged” by friction with an artificial polyester fur strip to achieve a saturated surface charge. However, the NC-Mode CCR-TENG experiences continuous charge decay during operation. To address this issue, the CCR-TENG employs a ternary dielectric soft-contact friction mechanism. As demonstrated in Figure 1c, the upper friction interface consists of PTFE film and nylon film. At the same time, a soft brush serves as the triboelectric medium layer on the lower copper electrode, utilizing polyester fur material. As depicted in Figure 1d, the NC-Mode CCR-TENG’s working principle involves charge generation, a transfer state, charge transfer and a stable state. When the polyester fur transitions from the initial state (i) to nylon, it comes into contact with and frictionally charges the nylon film. Due to the higher electronegativity of the polyester fur compared to the nylon film [31], the friction between them causes the separation of positive and negative charges, resulting in the nylon film carrying positive charges. Still, the polyester fur has negative charges (ii). As the turntable rotates, the polyester fur comes into frictional contact with the more electronegative PTFE film, generating new frictional charges (iii), which, along with the existing electrons on the polyester fur, are transferred to the PTFE film. Until the next piece of nylon film contacts the polyester fur, this will cause the PTFE film to acquire more electrons (iv), which is a crucial factor enabling the CCR-TENG to output high voltage [32]. With the turntable’s rotation, the next piece of nylon film frictionally contacts the polyester fur and acquires positive charges through charge transfer (v). According to the electrostatic induction effect, the charges on the electrodes redistribute between the two sets of electrodes via an external load to balance the potential difference. After cycling (ii–v), the charges on the PTFE and nylon film surfaces reach saturation (vi).

After frictional charging of the dielectric surface, air breakdown may occur between the two dielectrics due to the air gap. The maximum charge density is then present at the surface of the electrode [33]:

(2) $$Q_{max}=V_{\mathrm{gap}}\cdot C=V_{\mathrm{gap}}\cdot \frac{\varepsilon_{0}s}{d}$$

According to the capacitance model of the freestanding triboelectric-layer mode of the TENG [34], the output short-circuit current of the CCR-TENG can be expressed as:

(3) $$I=\frac{dQ}{dt}=A\frac{d\sigma }{dt}$$

Here, $V_{\mathrm{gap}}$ represents the potential difference between the dielectrics, $Q_{max}$ is the maximum charge density on the electrode surface, $\varepsilon_{0}$ denotes the vacuum permittivity constant and s denotes the electrode total area, d is the air gap, fixed at 3 mm. A and σ represent the effective contact area and surface charge density.

When charge transfer reaches saturation, an increase in wind speed boosts the turbine’s rotation speed and shortens the duration of each charge transfer cycle while the contact area remains unchanged. It implies that CCR-TENG achieves higher short-circuit current with increasing rotation speed at charge transfer saturation, but voltage and charge are unaffected by frequency. The unique design of the coaxial counter-rotating structure enhances the TENG’s low-speed startup performance, rotating performance and electrical output. To better exhibit the performance of the CCR-TENG, the startup wind speed, rotational speed, and power density per wind speed unit are compared with related designs in Figure 1e–g. The CCR-TENG is superior in all three comparisons, and the detailed parameters are recorded in Table S1. In Figure 1e, startup wind speeds in references range from 1 to 4.5 m/s, and the startup wind speed for the CCR-TENG with NC-Mode is only 1 m/s [22,35,36,37,38,39,40]. As depicted in Figure 1f, at a wind speed of 5 m/s, the CCR-TENG achieves a rotational speed of 420 rpm with SC-Mode, while none of the references exceed 250 rpm [22,35,37,38,39,40]. In Figure 1g, normalized power density at different wind speeds is compared with previous works, showing that the power density per wind speed of the CCR-TENG is 746 mW/m^3^·s/m, which is more than three times the highest value in the references [22,35,36,37,38,39,40]. In the comparison, the CCR-TENG has the best performance of startup wind speed, rotation speed, and electrical output performance.

### 3.2. Blade Selection and Structural Optimization

**Figure 2:**
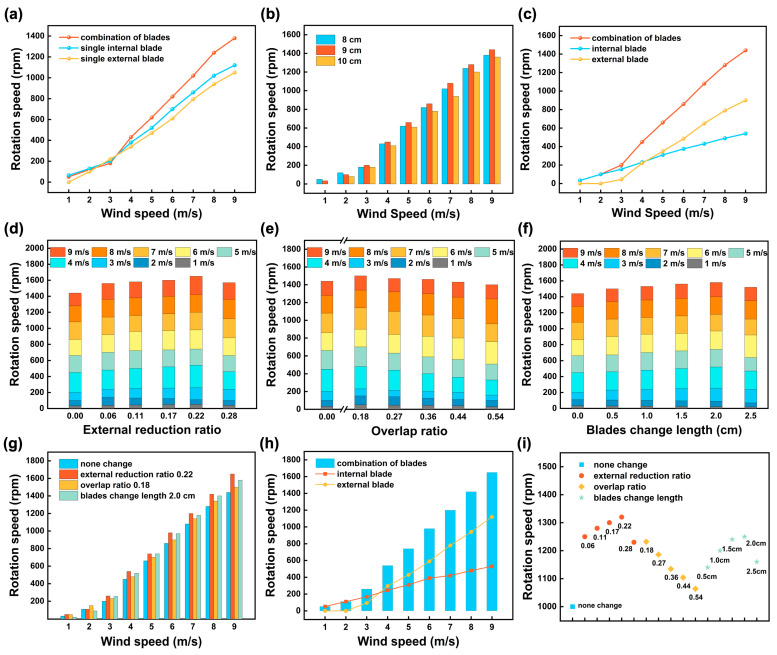
Selection and structural optimization of wind turbine blades. (**a**) Comparison of the relative speeds of single internal and external blades and their combination; (**b**) The relative speed of the CCR-TENG with different chord lengths of external blades; (**c**) Distribution of speeds of internal and external blades when combined in CCR-TENG rotation; (**d**) Influence of internal blade external reduction ratio on the relative speed of the CCR-TENG; (**e**) Influence of overlap ratio of internal blades on the relative speed of the CCR-TENG; (**f**) Influence of internal blade external reduction and overlap on the relative speed of the CCR-TENG; (**g**) Comparison of relative speeds of the CCR-TENG under different structural optimization methods for internal blades; (**h**) Comparison of the relative rotational speeds of the CCR-TENG, and the rotational speeds of the internal and external blades; (**i**) Structural selection of SC-Mode for different CCR-TENG combinations.

For better startup and rotation performance of the TENG, a combination of S1046 airfoil-type external blades and traditional Savonius drag-type internal blades was chosen to fabricate the CCR-TENG [41,42]. Considering manufacturing cost and wind energy utilization efficiency, a three-blade structure was chosen for the airfoil-type knives. At the same time, the traditional two-blade design was selected for the drag-type blades. The blade selection and structural optimization process of the wind turbine of the CCR-TENG are investigated and shown in Figure 2. Figure 2a analyzes the rotational speed variation after combining the internal and external blades of the CCR-TENG. The relative rotational speed of the coaxial counter-rotating blades is higher than that of the individual internal and external blades but significantly lower than the sum of their individual rotational speeds. The results demonstrate a significant interaction between the internal and external blades during operation in the coaxial counter-rotating mode. This interaction needs to be reduced to optimize performance, as it is not feasible to arbitrarily combine lift and drag blades. Further experimental research is needed to explore the structural optimization of the CCR-TENG to leverage its advantages fully. Figure S1a further demonstrates the rotational speed of the internal and external blades during coaxial counter-rotating. After combination, the internal blades exhibit better low-speed startup performance, achieving low-speed start at 1 m/s wind velocity, while the external blades startup and achieve coaxial counter-rotating only after 3 m/s. At wind velocities exceeding 6 m/s, the rotational speed of the external blades surpasses that of the internal blades, indicating superior rotational performance of the external blades under higher wind velocities.

For single-blade TENG, the blade’s chord length is considered a crucial factor in enhancing the startup performance of the external blade. An increase in the chord length increases the blade thickness and static moment coefficient, making it easier for the wind turbine to achieve low-speed startup [43]. External blades with chord lengths of 8 cm, 9 cm, and 10 cm were separately produced and combined with internal blades to form CCR-TENGs of different specifications. The relative rotation speeds of the CCR-TENGs were measured, and the experimental results in Figure 2b indicate that the CCR-TENG achieves the highest relative rotation speed when the external blade chord length is 9 cm. Figure S1b,c show the rotation speeds of the external and internal blades of CCR-TENGs with different chord lengths under various wind conditions. Increasing the chord length of the external blade reduces the startup wind speed and increases its rotation speed. Still, it may also reduce the direct wind-receiving area of the internal blade, significantly decreasing its rotation speed. When the external blade chord length is 9 cm, the CCR-TENG achieves the highest relative rotation speed. Figure 2c illustrates the relative rotational speeds of the CCR-TENG with an external blade chord length of 9 cm and the distribution of speeds between the internal and external blades. The internal blade can start rotating at 1 m/s and achieves a speed of 33 rpm, while the external blade starts spinning at 3 m/s and reaches a speed of 45 rpm. Beyond 4 m/s, the rotational speed of the external blade exceeds that of the internal blade. The internal blades are optimized to mitigate the mutual influence of the internal and external blades during coaxial counter-rotating and increase the relative rotation speed.

As shown in Figure 2d–f, the optimized internal blade and the 9 cm chord length external blade formed CCR-TENGs of different specifications. There are 16 sets of CCR-TENGs with different structures. The CCR-TENG, where the external blade chord length is 9 cm and the internal blade placement is not optimized, is defined as the original CCR-TENG, corresponding to the cases of Figure 2d–f, where the change in the horizontal coordinate is zero. As shown in Figure S1d, the internal blades are made with external reduction. Figure 2d demonstrates the relationship between the relative rotation speed of the CCR-TENGs with different external ratio of reduction and wind speed. The relative rotation speed with external ratio reduction shows a pattern of initial ascent followed by a decline, with the highest relative rotation speed at an external reduction ratio of 0.22. At 9 m/s, the rotation speed increased from 1440 rpm (no reduction) to 1650 rpm. External reduction reduces the moment of inertia of the internal blade, resulting in lower resistance under the same wind conditions and demonstrating higher rotation speed. At the same time, the mutual influence between the internal and external blades is reduced. As shown in Figure S1e, the external blade rotational speed of the CCR-TENG after the external reduction is improved compared with that of the original CCR-TENG external blade and increases with the increase of the retraction ratio.

As shown in Figure S1f, the internal blade rotational speed of the CCR-TENG increases and then decreases with the rise of the retraction ratio after the external reduction. At the time of retraction 0.28, the external blade rotational speed is lower than that of the original CCR-TENG external blade, mainly due to the external blade’s rapidly increasing rotation speed affecting the internal blade’s rotation. Researchers generally consider a blade overlap ratio of 0.15–0.20 optimal for the static startup and power performance of Savonius blades [44,45]. Figure S1g shows the demonstration of internal blade overlap treatment. Figure S1h,i show the variation in the rotation speed of the internal and external blades of the CCR-TENG under different wind conditions when an overlap ratio exists. The rotation speed of the internal blade shows a pattern of initial ascent followed by a decline with the increase in the overlap ratio, with the most significant high at 0.18. However, the rotation speed of the external blade decreases with an increasing overlap ratio. Figure 2e demonstrates the relative rotation speed of the CCR-TENGs with different overlap ratio as a function of wind speed. The CCR-TENG exhibits the optimal startup and rotation performance when the overlap ratio is 0.18. Under a wind speed of 1 m/s, CCR-TENG can start and further increase its rotational speed by 17 rpm. Moreover, it enhances the relative rotation speed to reach a maximum speed of 1500 rpm at a wind speed of 9 m/s. Figure 2f investigates the effect of external reduction and internal overlap of the same size on the rotation speed of the CCR-TENG. Under a wind condition of 9 m/s, the relative rotation speed of the CCR-TENG with 2 cm external reduction and internal overlap is 1580 rpm, higher than the relative rotation speed of the original CCR-TENG (1440 rpm) but lower than that of CCR-TENG with an external reduction ratio of 0.22 (1650 rpm).

Among the four structures, external reduction, internal overlap, external reduction with internal overlap, and the original CCR-TENG, the external reduction ratio of 0.22 exhibits the highest relative rotation speed, as shown in Figure 2g. Figure 2h demonstrates the relative rotation speed of the CCR-TENG with an external reduction ratio of 0.22 and the ratio of the rotation speeds of the internal and external blades during coaxial counter-rotating as a function of wind speed. The internal blade can start at 1 m/s wind speed, while the external blade starts rotating at 3 m/s and achieves coaxial counter-rotating. After 3 m/s, the rotation speed of the external blade exceeds that of the internal blade. Since the results are obtained in NC-Mode, the non-connected CCR-TENG with an external reduction ratio of 0.22 is called the NC-Mode CCR-TENG, and this will be used for subsequent electrical energy testing in NC-Mode. It transitions into SC-Mode by attaching one piece of polyester fur strip to the rotating turntable of the NC-Mode CCR-TENG. Figure 2i shows soft-contact friction structure selection for the above 16 CCR-TENGs at 8 m/s wind speed. Among the different CCR-TENGs, the performance of the CCR-TENG with an external reduction ratio of 0.22 is optimal. Therefore, this CCR-TENG, which undergoes triboelectric friction with a ternary dielectric and has an external reduction ratio of 0.22, is called the soft-contact mode (SC-Mode) CCR-TENG. The structure will be used for subsequent electrical energy testing in SC-Mode. The original TENG consists of the non-optimized internal blade and the external turntable of an unassembled S1046 airfoil-type external blade, and this structure will be used in the comparison to derive the NC-mode performance improvement ratio of the CCR-TENG.

### 3.3. Electrical Output of the NC-Mode CCR-TENG

**Figure 3:**
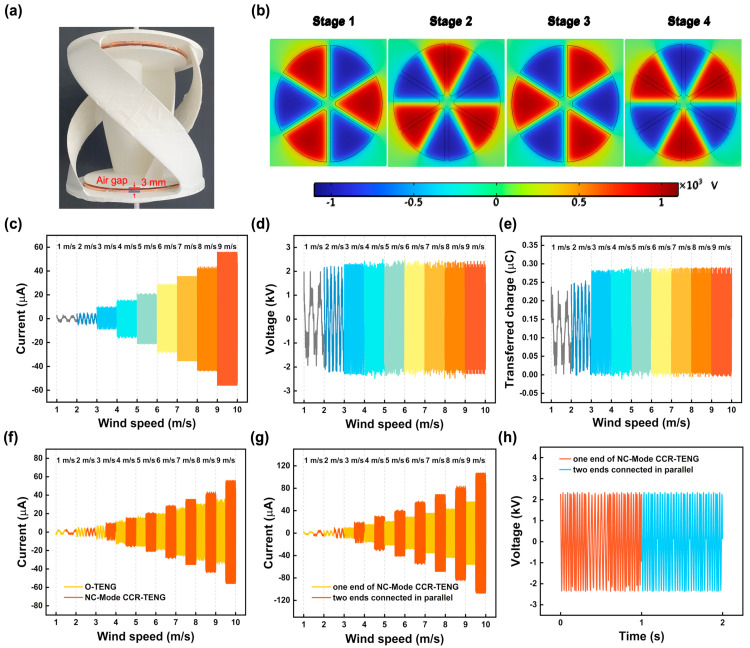
Electrical output of the NC-Mode CCR-TENG. (**a**) Practical demonstration of the NC-Mode CCR-TENG; (**b**) Electrical potential simulation of the NC-Mode CCR-TENG; (**c**) Current graph of the NC-Mode CCR-TENG; (**d**) Voltage graph; (**e**) Transferred charge graph; (**f**) Comparison of current between the NC-Mode CCR-TENG and the NC-Mode original TENG; (**g**) Comparison of current at both ends of the CCR-TENG before and after parallel connection; (**h**) Comparison of voltage at both ends of the CCR-TENG before and after parallel connection.

The impact of the coaxial counter-rotating structure was assessed by the electrical output of the CCR-TENG in NC-Mode within a low-speed wind tunnel. Figure 3a shows the structure of the NC-Mode CCR-TENG with an air gap of 3 mm. Figure 3b presents an electrical potential simulation of the NC-Mode CCR-TENG, indicating an estimated potential difference of approximately 2.2 × 10^3^ V between adjacent electrodes. Figure S2 shows the simulation of charge density for the NC-Mode CCR-TENG. The simulation results suggest that the charge density is approximately 13.5 × 10^−6^ C/m^2^. Both of the above results are simulation results based on experimental conditions. Figure 3c–e illustrate the relationships between $I_{SC}$, $V_{OC}$, $Q_{SC}$, and wind speed for the NC-Mode CCR-TENG. $I_{SC}$ increases with increasing wind speed. $V_{OC}$ and $Q_{SC}$ exhibit similar trends, initially improving and stabilizing with higher wind speeds, and reaching stability at 3 m/s inlet wind speed. The maximum induced charge density and the corresponding maximum potential difference occur at 3 m/s, $V_{OC}$, stabilizing at 2.2 kV, in Figure 3d, and $Q_{SC}$ at 0.28 µC in Figure 3e. The contact area remains constant, while increasing the wind speed shortens each charge transfer cycle. As a result, $I_{SC}$ continues to grow with higher wind speeds, reaching 50 µA at 9 m/s for the CCR-TENG, as shown in Figure 3c.

Figure 3f compares short-circuit current output of the NC-Mode original TENG and the NC-Mode CCR-TENG at different wind speeds. At 9 m/s wind speed, the NC-Mode CCR-TENG achieves an $I_{SC}$ of 50 µA, while the NC-Mode original TENG only reaches 35 µA, the coaxial counter-rotating structure of the CCR-TENG enhances short-circuit current output by 42.9%. It is found that short-circuit current of the CCR-TENG only became noticeably higher than the original TENG when the wind speed exceeded 3 m/s. This conclusion is because, at wind speeds below 3 m/s, both devices only rotate at low speeds, resulting in a relatively small short-circuit current. Even though the coaxial reverse structure can enhance the relative rotation speed of the TENG, its effect is limited when the wind speed is below 3 m/s. Figure 3g compares short-circuit current output before and after the parallel connection of two TENGs in the CCR-TENG. Due to device fabrication limitations, the parallel CCR-TENG exhibits a slightly lower $I_{SC}$ than the twice short-circuit current of the single-blade CCR-TENG, with a maximum of 0.098 mA. Figure 3h shows the voltage changes with wind speed before and after the parallel connection of two TENGs in the CCR-TENG, with no change in voltage values. It can be concluded that the NC-Mode CCR-TENG exhibits good startup and rotation performance, especially as it can start at an ultra-low wind speed of 1 m/s. However, during the electrical output experiments, it was observed that the friction charges of the NC-Mode CCR-TENG tend to decay easily, resulting in an unstable electrical output.

### 3.4. Electrical Output of SC-Mode CCR-TENG

**Figure 4:**
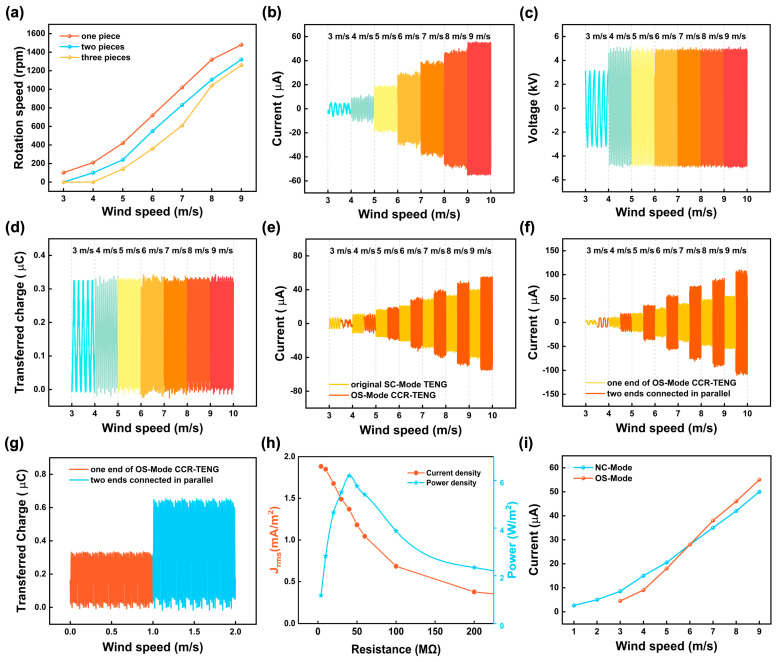
Electrical output of the SC-Mode CCR-TENG.(**a**) Influence of the number of polyester fur strips on the rotational speed of the CCR-TENG; (**b**) Current graph of the OS-Mode CCR-TENG at different inflow wind speeds; (**c**) Voltage graph; (**d**) Transferred charge graph; (**e**) Current graph of the OS-Mode CCR-TENG compared to the original SC-Mode TENG at different wind speeds; (**f**) Comparison of current at both ends of the OS-Mode CCR-TENG before and after parallel connection; (**g**) Comparison of transferred charge at both ends of the OS-Mode CCR-TENG before and after parallel connection; (**h**) The J_rms_ and power of the OS-Mode CCR-TENG with different load resistances (2 MΩ to 225 MΩ); (**i**) Comparison of current between the OS-Mode CCR-TENG and the NC-Mode CCR-TENG.

During experiments, changes in electrical performance are observed for the NC-Mode CCR-TENG due to charge decay. In contrast, the SC-Mode CCR-TENG is expected to have a constant electrical performance when exposed to a fixed wind speed. Experiments were conducted in a low-speed wind tunnel to attain the electrical output of the SC-Mode CCR-TENG. Figure 4 investigates the electrical output performance of CCR-TENG with different influencing factors. Figure 4a indicates that CCR-TENG outfitted with one polyester fur strip exhibits improved startup and rotational performance. Figure S3a,b further demonstrate that an increment in the number of polyester fur strips significantly impedes the ability of both the internal and external blades to rapidly increase their rotational speed within the wind velocity range of 3–5 m/s, suggesting that a larger quantity of polyester fur strips diminishes the CCR-TENG’s rotational efficacy and impedes the startup. The evidence from both figures consistently supports the superior startup and rotational behavior of the CCR-TENG when one polyester fur strip strand is employed. Figure S3c compares the variations in electrical short-circuit current output of the CCR-TENGs with varying numbers of polyester fur strips under different wind speeds. One polyester fur strip generates higher short-circuit current output, up to 56 µA. The SC-Mode CCR-TENG with polyester fur strip has subsequently been called the optimal soft-contact mode (OS-Mode) CCR-TENG. The OS-Mode CCR-TENG will be used to conduct the subsequent electrical performance experiments.

Figure 4b–d depict the relationships of $I_{SC}$, $V_{OC}$, and $Q_{SC}$ with wind speed for the OS-Mode CCR-TENG. $I_{SC}$ increases with wind speed, while $V_{OC}$ and $Q_{SC}$ increase up to a wind speed of 4 m/s and remain essentially constant thereafter. At the maximum experimental wind speed of 9 m/s, the OS-Mode CCR-TENG achieves $I_{SC}$ of 56 µA, $V_{OC}$ of 4.9 kV, and $Q_{SC}$ of 0.33 µC. Adding a polyester fur strip to the original TENG and employing ternary dielectric soft-contact friction is called the original SC-Mode TENG. Figure 4e shows the variation of short-circuit current output with wind speed for the OS-Mode CCR-TENG versus the original SC-Mode TENG. At a wind speed of 9 m/s, the $I_{SC}$ of the original SC-Mode TENG is 39 µA, while the $I_{SC}$ of the OS-Mode CCR-TENG is 56 µA, which is an improvement of 41%. The OS-Mode CCR-TENG has a higher short-circuit current output but lower startup performance than the original SC-Mode TENG below 4 m/s wind speed. Still, due to the addition of polyester fur, it can only be initiated at wind speeds of 3 m/s and above. Figure 4f compares short-circuit current variation with wind speed before and after paralleling the two TENGs at both ends of the OS-Mode CCR-TENG. The $I_{SC}$ of the CCR-TENG after the paralleling was connected almost doubles compared with the $I_{SC}$ of the single-side OS-Mode CCR-TENG, which can be up to 0.11 mA. Figure 4g shows the comparison of the transferred charge before and after the paralleling of the TENG at both ends of the OS-Mode CCR-TENG, and the $Q_{SC}$ of the CCR-TENG after paralleling can reach 0.64 µC.

To systematically investigate the power output of the SC-Mode CCR-TENG, an external high-voltage resistor bar is used as a load resistance to analyze and compare the power output of the OS-Mode CCR-TENG. The peak power of the SC-Mode CCR-TENG at a wind speed of 9 m/s is obtained. The voltage and short-circuit current measured under variable external resistance (10 MΩ to 475 MΩ) are shown in Figure S4. The short-circuit current decreases with increasing resistance, while the voltage shows the opposite trend. The power increases in the low-resistance stage and falls in the high-resistance stage. At around 40 MΩ, the maximum power is 256 mW, the peak surface power density is 6.2 W/m^2^. The peak volumetric power density per unit wind speed is 746 mW/m^3^·s/m, as shown in Table S1. At the same time, plotting the correlation between root mean square current density (J_rms_) current density and power is necessary to prevent the effect of peak current on the CCR-TENG power output plot [46]. The J_rms_ and power of the OS-Mode CCR-TENG with different load resistances (2 MΩ to 225 MΩ) are shown in Figure 4h at 9 m/s wind speed. The variation of short-circuit current with wind speed for the NC-Mode CCR-TENG and the OS-Mode CCR-TENG is shown in Figure 4i. Incorporating polyester fur increases the rotational resistance of the CCR-TENG, resulting in the OS-Mode CCR-TENG being able to startup when the wind speed reaches 3 m/s. Additionally, in the wind speed range of 3–6 m/s, it does not exhibit a higher electrical short-circuit current output than the NC-Mode CCR-TENG. With the increase in wind speed, the OS-Mode CCR-TENG gains more energy to counteract the friction from the polyester fur, progressively showing a higher electrical short-circuit current output than its NC-Mode counterpart when wind speed reaches 6 m/s. It means that each of the two modes of the CCR-TENG has advantages, the NC-Mode CCR-TENG is more suitable for collecting energy from ultra-low wind speeds than the SC-Mode CCR-TENG. Stable output is a requirement for the CCR-TENG to realize the in-situ energy supply of distributed WSNs, and a long-term durability experiment can better evaluate the durability of the TENG [47]. As shown in Figure S5, the durability test of the OS-Mode CCR-TENG is conducted under 4 m/s wind speed, and the output remains unchanged after 750 k rotation cycles.

### 3.5. Demonstration of the CCR-TENG

**Figure 5:**
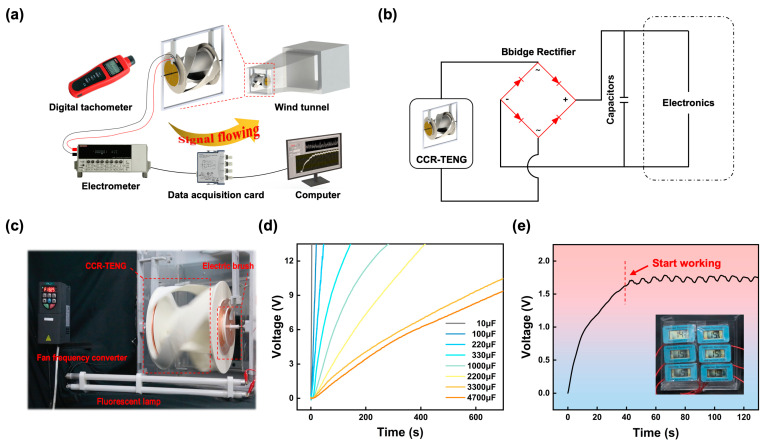
Demonstration of the CCR-TENG as a sustainable energy source. (**a**) Experimental system diagram; (**b**) Circuit diagram of using the CCR-TENG as a sustainable power source for electronic devices; (**c**) Demonstration of the CCR-TENG powering two fluorescent lamps; (**d**) Charging curves of the CCR-TENG for eight different commercial capacitors; (**e**) Demonstration of the OS-Mode CCR-TENG continuously powering six temperature sensors.

The application of the CCR-TENG is demonstrated through a series of experiments. Figure 5a depicts the experimental setup in a low-speed wind tunnel, with the CCR-TENG positioned vertically at the center of the tunnel inlet to capture the incoming airflow. Figure 5b illustrates the circuit diagram for utilizing the CCR-TENG as a sustainable power source to supply power to electronic devices continuously. Figure S6 shows the specific dimensions of the experimental wind tunnel in three views. The electrical signal generated by the CCR-TENG is rectified through a bridge rectifier circuit, charging a commercial capacitor and then powering the electronic device. Figure 5c showcases the capability of the OS-Mode CCR-TENG to illuminate two 14 W fluorescent lamps at 4 m/s wind speed, with the video demonstration provided in Video S2. Video S3 shows an experiment with the OS-Mode CCR-TENG powering 100 series-connected LEDs each rated at 36 V when the wind speed is 4 m/s. The two videos show that the CCR-TENG can enable in-situ energy supply for WSNs at low wind speeds. The TENGs at both ends of the OS-Mode CCR-TENG are connected in parallel to continuously charge eight different commercial capacitors, as shown in Figure 5d. The charging results demonstrate that the CCR-TENG can continuously charge capacitors of different specifications and maintain a high voltage output, making it possible for in-situ powering for WSNs. Figure 5e depicts the ability of the OS-Mode CCR-TENG, under stable wind speed conditions of 6 m/s, to power six temperature sensors continuously after charging a 330 µF capacitor for 42 s, with the video demonstration of the sustained power supply for the temperature sensors provided in Video S4. The CCR-TENG has sufficient capacity to power more WSNs at high wind speeds.

## 4. Conclusions

A coaxial counter-rotating triboelectric nanogenerator with lift-drag hybrid blades, termed a CCR-TENG, has been proposed to harvest low-speed wind energy efficiently. Capable of operating in both SC-Mode and NC-Mode, the CCR-TENG utilizes distinct structural designs for its internal and external blades to achieve the coaxial counter-rotating effect. Compared to single-blade TENGs, the coaxial counter-rotating design of the CCR-TENG reduces the overall structural resistance encountered by the blades during high-speed rotation, enhancing the startup and rotational performance and subsequently improving the electrical output of the CCR-TENG. Experiments were conducted to reveal further the impact of variables such as the chord length of the external blades, structural design of the internal blades, the optimum number of polyester fur strips in SC-Mode, and the effect of wind speed on the startup performance, rotational performance, and electrical output of the CCR-TENG. Through the structure selection experiments, a CCR-TENG with an external blade chord length of 9 cm and an internal blade external reduction ratio of 0.22 exhibited superior performance, making it the chosen base structure for both NC-Mode and SC-Mode in electrical performance experiments.

The NC-Mode CCR-TENG effectively harvests wind energy at speeds of 1 m/s and above, reaching a short-circuit current output of 0.098 mA at 9 m/s, although it experiences charge decay in this mode. In contrast, the SC-Mode offers stable and higher performance output. The SC-Mode CCR-TENG achieves a maximum short-circuit current of 0.11 mA, a peak power of 256 mW, and a peak surface power density of 6.2 W/m^2^, with a unit wind speed peak volumetric power density of 746 mW/m^3^·s/m. Compared to the original SC-Mode TENG, the OS-Mode CCR-TENG shows a 41% increase in short-circuit current output under the same inflow conditions. The OS-Mode CCR-TENG continuously illuminates two fluorescent lamps at a fixed low wind speed of 4 m/s, and six temperature sensors are continuously lit at 6 m/s. This result means that it can supply WSNs with energy in situ. Most importantly, the emergence of the CCR-TENG provides a viable solution for on-site energy supply to distributed WSNs in the IoT era, and it is expected to achieve in-situ energy supply for many distributed WSNs through wind energy harvesting.

## Data Availability

Data are contained within the article.

## Supplementary Materials

The following supporting information can be downloaded at: https://www.mdpi.com/article/10.3390/nano14070598/s1, Table S1: Comparison and summary of the output performances, rotation performance and wind speed of previous rotation triboelectric nanogenerators for harvesting wind energy; Note S1: Formulas for calculation of external reduction ratio and overlap ratio; References [22,35,36,37,38,39,40] are cited in the supplementary materials; Figure S1: Selection and structural optimization of turbine blades; Figure S2: Charge density simulation graph for the NC-Mode CCR-TENG; Figure S3: Rotation speed and output at different numbers of polyester fur strips; Figure S4: Power matching graph of OS-Mode CCR-TENG; Figure S5: The durability test of OS-Mode CCR-TENG; Figure S6: The specific dimensions of the experimental wind tunnel in three views; Video S1: Demonstration of the CCR-TENG coaxial counter-rotating effect at 0.5 times recording speed; Video S2: The CCR-TENG lighting display; Video S3: The OS-Mode CCR-TENG lights up 100 commercial LEDs; Video S4: Demonstration of the soft-connect CCR-TENG continuously powering six temperature sensors.
