# Supplementary material for: High-Performance Coaxial Counter-Rotating Triboelectric Nanogenerator with Lift–Drag Hybrid Blades for Wind Energy Harvesting
Source: Nanomaterials (Basel). 2024 Mar 28;14(7):598. doi: 10.3390/nano14070598 (PMC11013478; doi:10.3390/nano14070598)
Supplement: Supplementary file 1 [file nanomaterials-14-00598-s001.zip › nanomaterials-2900035-supplementary.pdf]

High-Performance Coaxial Counter-Rotating Triboelectric Nanogenerator with Lift–Drag Hybrid Blades for Wind Energy Harvesting

Fei Yan [1,†], Junhao Zhao [1,*,†], Fangming Li [1,*,†], Yiyao Chu [1], Hengxu Du [1], Minzheng Sun [1], Ziyue Xi [1], Taili Du [1,2] and Minyi Xu [1,3,*]

1 Dalian Key Lab of Marine Micro/Nano Energy and Self-Powered System, Marine Engineering College, Dalian Maritime University, Dalian 116026, China; yf1169@dlmu.edu.cn (F.Y.); chuyiyao@dlmu.edu.cn (Y.C.); duhengxu@foxmail.com (H.D.); sunminzheng@dlmu.edu.cn (M.S.); yyds@dlmu.edu.cn (Z.X.); dutaili@dlmu.edu.cn (T.D.)
2 Collaborative Innovation Research Institute of Autonomous Ship, Dalian Maritime University, Dalian 116026, China
3 State Key Laboratory of Maritime Technology and Safety, Dalian 116026, China
* Correspondence: haoger@dlmu.edu.cn (J.Z.); lifangming@dlmu.edu.cn (F.L.); xuminyi@dlmu.edu.cn (M.X.)
† These authors contribute equally to this work.

Table S1. Comparison of the output performances, rotation speed and wind speed of previous rotation triboelectric nanogenerators for harvesting wind energy.

	Working wind speed range (m/s)	Wind speed (m/s)	Peak power of TENG/EMG (mW)	Device volume (mm³)	Peak power density of TENG（W/m³）	Peak power density per wind speed (mW/m³·s/m）	Rotation speed when wind speed is 5m/s△
**1**	2.6-6	6	0.38/118	3042000	0.13	21	160
**2**	2.2-16	6	2/0	11451105	0.18	29	230
**3**	2-12	12	2.79/0	3958407	0.71	59	200
**4**	1-7	3	4/0	5089380	0.79	262	100
**5**	4-21	10	7/21	5340708	1.31	131	95
**6**	2-10	10	24.7/620	14137167	1.75	175	-
**7**	4.5-19	13	22.85/6.28	6480000	3.53	272	38
**This work**	1-9	9	256/0	38168779	6.71	746	420

△ Partial data is not given in the references, and is calculated according to the size of the prototype. At the wind speeds shown in the table, each study has a corresponding peak power of TENG/EMG, peak power density of TENG, and peak power density per wind speed.

References

1. Gui, Y.; Wang, Y.; He, S.; Yang, J. Self-Powered Smart Agriculture Real-Time Sensing Device Based on Hybrid Wind Energy Harvesting Triboelectric-Electromagnetic Nanogenerator. Energy Conversion and Management 2022, 269, 116098.
2. Yong, S.; Wang, J.; Yang, L.; Wang, H.; Luo, H.; Liao, R.; Wang, Z.L. Auto-Switching Self-Powered System for Efficient Broad-Band Wind Energy Harvesting Based on Dual-Rotation Shaft Triboelectric Nanogenerator. Advanced Energy Materials 2021, 11, 2101194.
3. Zou, H.-X.; Zhao, L.-C.; Wang, Q.; Gao, Q.-H.; Yan, G.; Wei, K.-X.; Zhang, W.-M. A Self-Regulation Strategy for Triboelectric Nanogenerator and Self-Powered Wind-Speed Sensor. Nano Energy 2022, 95, 106990.
4. Liu, D.; Li, C.; Chen, P.; Zhao, X.; Tang, W.; Wang, Z.L. Sustainable Long-Term and Wide-Area Environment Monitoring Network Based on Distributed Self-Powered Wireless Sensing Nodes. Advanced Energy Materials 2023, 13, 2202691.
5. Li, X.; Gao, Q.; Cao, Y.; Yang, Y.; Liu, S.; Wang, Z.L.; Cheng, T. Optimization Strategy of Wind Energy Harvesting via Triboelectric-Electromagnetic Flexible Cooperation. Applied Energy 2022, 307, 118311.
6. Zhu, M.; Yu, Y.; Zhu, J.; Zhang, J.; Gao, Q.; Li, H.; Zhang, Y.; Wang, Z.L.; Cheng, T. Bionic Blade Lift-Drag Combination Triboelectric-Electromagnetic Hybrid Generator with Enhanced Aerodynamic Performance for Wind Energy Harvesting. Advanced Energy Materials n/a, 2303119.
7. Dang, H.; Wang, Y.; Zhang, S.; Gao, Q.; Li, X.; Wan, L.; Wang, Z.L.; Cheng, T. Triboelectric-Electromagnetic Hybrid Generator with the Inertia-Driven Conversion Mechanism for Wind Energy Harvesting and Scale Warning. Materials Today Energy 2022, 29, 101136.

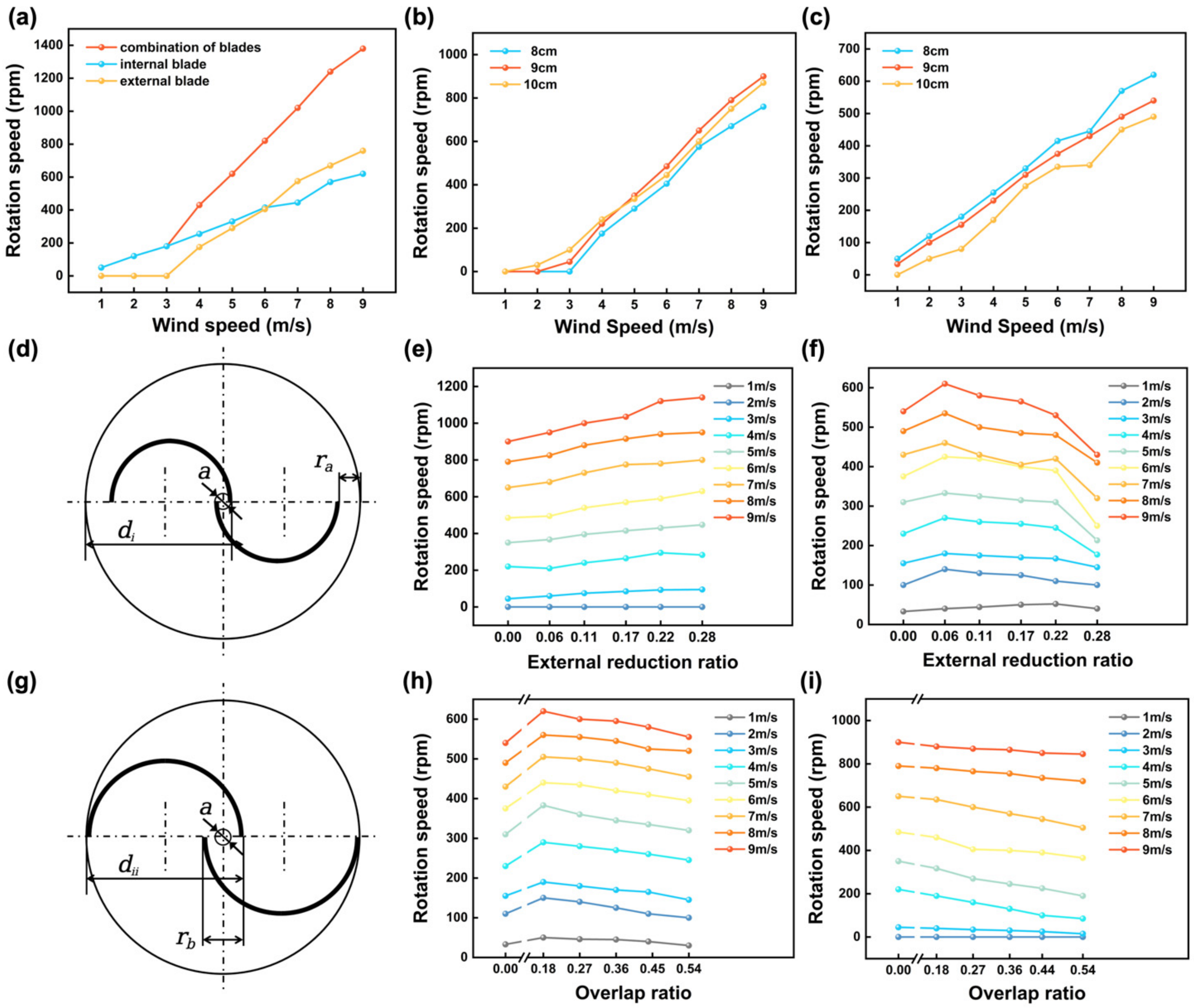

Figure S1. Selection and structural optimization of turbine blades. **(a)** Rotational speed of internal and external blades

during coaxial reversal; **(b)** External blade rotational speed of CCR-TENG with different chord lengths; **(c)** Internal blade rotational speed of CCR-TENG with different chord lengths; **(d)** Demonstration of internal blade external reduction treatment; **(e)** External blade speed of CCR-TENG after external reduction; **(f)** Internal blade speed of CCR-TENG after external reduction; **(g)** Demonstration of internal blade overlap treatment; **(h)** Internal blade speed of CCR-TENG after overlap; **(i)** External blade speed of CCR-TENG after overlap.

Note S1:

Figure S1d shows a schematic diagram of the internal blade made to be externally reduced, where the equation gives the external reduction ratio.

$$i = \frac{r_a}{d_i} \tag{S1}$$

In the formula, d_i represents the blade diameter (including the shaft diameter a), and r_a represents the external reduction length.

Figure S1g shows a schematic diagram of the internal blade undergoing overlap treatment, where the formula for blade overlap ratio is provided.

$$\beta = \frac{r_b - a}{d_{ii}} \tag{S2}$$

In the formula, r_b represents the length of the overlapping, a represents the shaft diameter, and d_i represents the blade diameter (including the length of the overlapping r_b).

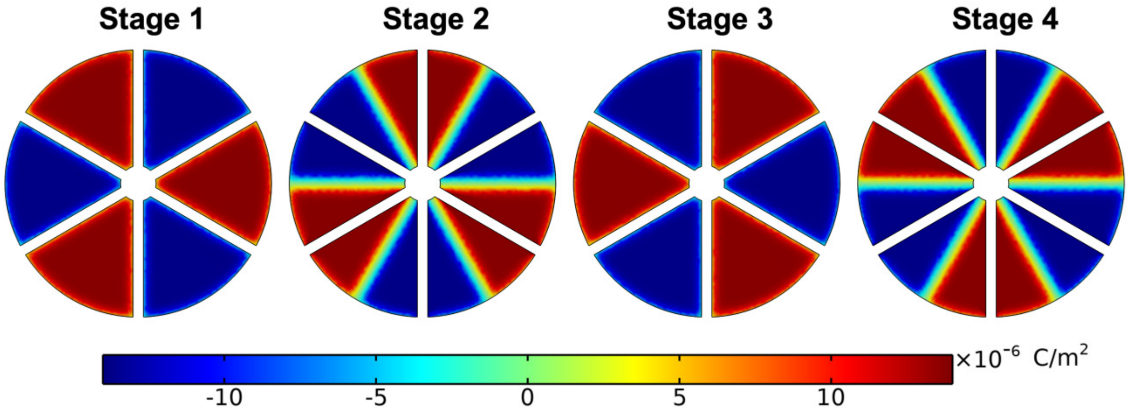

Figure S2. Charge density simulation graph for NC-Mode CCR-TENG.

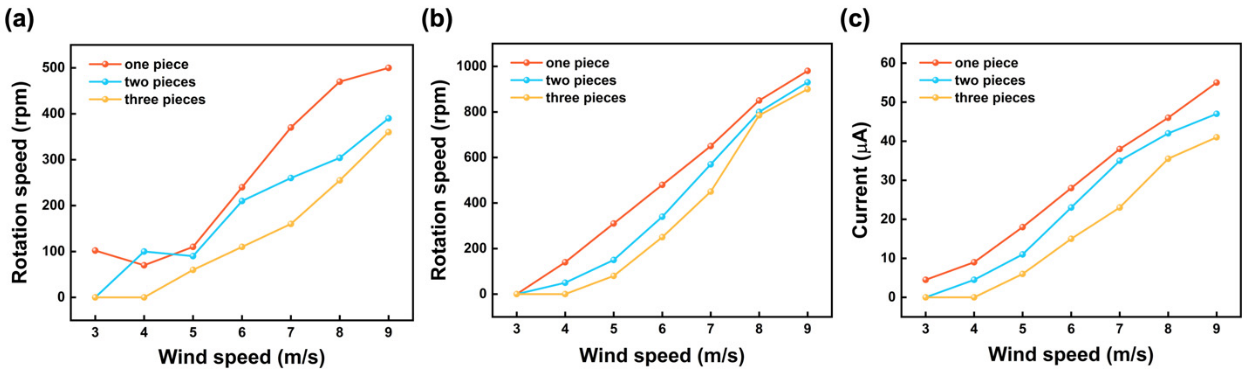

Figure S3. Rotation speed and output at different numbers of polyester fur strips. **(a)** Internal blade rotation speed of CCR-TENG with different numbers of polyester fur strips; **(b)** External blade rotation speed of CCR-TENG with different numbers of polyester fur strips; **(c)** Current of CCR-TENG with different numbers of polyester fur strips.

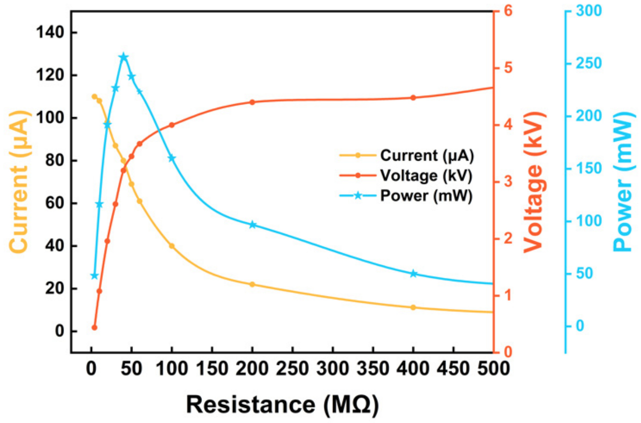

Figure S4. Power matching graph of OS-Mode CCR-TENG

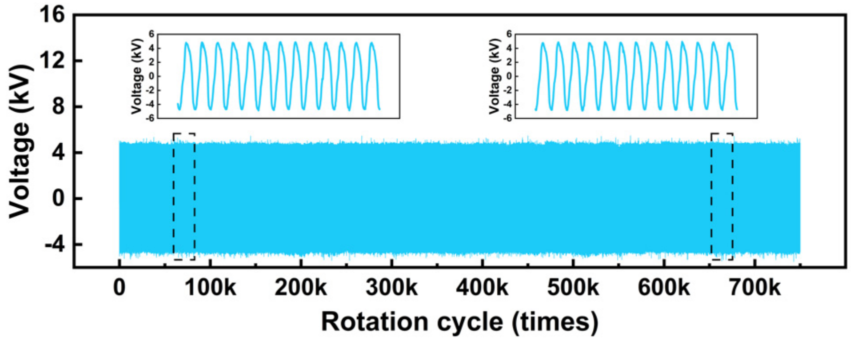

Figure S5. The durability test of OS-Mode CCR-TENG.

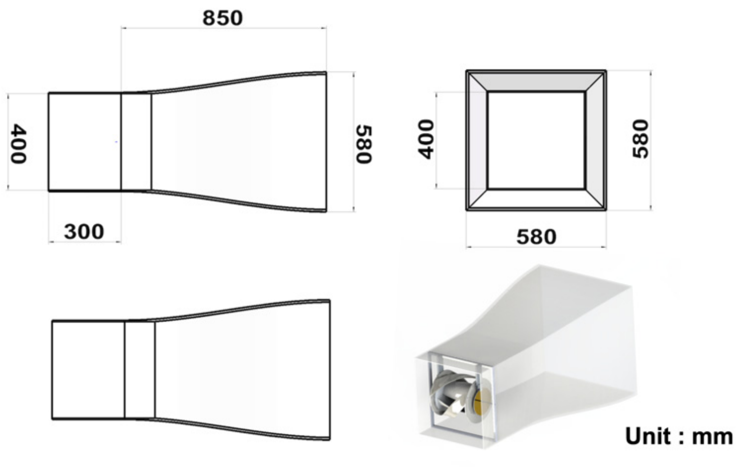

Figure S6. The specific dimensions of the experimental wind tunnel in three views.
